# Prevalence and characterization of IncQ1α-mediated multi-drug resistance in *Proteus mirabilis* Isolated from pigs in Kunming, Yunnan, China

**DOI:** 10.3389/fmicb.2024.1483633

**Published:** 2025-01-09

**Authors:** Hongmei Liu, Na Xia, Fanan Suksawat, Bundit Tengjaroenkul, Yue Hu, Xiaofeng Zhou, Xiaojiang Li, Cuiqin Huang, Yinli Bao, Qiong Wu, Chunrong Zhang, Sunpetch Angkititrakul, Bin Xiang, Xin Wu

**Affiliations:** ^1^Yunnan Joint International R&D Center of Veterinary Public Health, College of Veterinary Medicine, Yunnan Agricultural University, Kunming, China; ^2^Engineering Research Center for the Prevention and Control of Animal Original Zoonosis of Fujian Province University, College of Life Science, Longyan University, Fujian, China; ^3^Faculty of Veterinary Medicine, Khon Kaen University, Khon Kaen, Thailand

**Keywords:** *Proteus mirabilis*, antimicrobial resistance, Inc plasmid, *repC* gene, whole genome sequence

## Abstract

**Background:**

*Proteus mirabilis* is a conditionally pathogenic bacterium that is inherently resistant to polymyxin and tigecycline, largely due to antibiotic resistance genes (ARGs). These ARGs can be horizontally transferred to other bacteria, raising concerns about the Inc plasmid-mediated ARG transmission from *Proteus mirabilis*, which poses a serious public health threat. This study aims to investigate the presence of Inc plasmid types in pig-derived *Proteus mirabilis* in Kunming, Yunnan, China.

**Methods:**

Fecal samples were collected from pig farms across six districts of Kunming (Luquan, Jinning, Yiliang, Anning, Songming, and Xundian) from 2022 to 2023. *Proteus mirabilis* isolates were identified using *IDS* and *16S rRNA* gene sequencing. Then, positive strains underwent antimicrobial susceptibility testing and incompatibility plasmid typing. Multi-drug-resistant isolates with positive incompatibility plasmid genes were selected for whole-genome sequencing. Resistance and Inc group data were then isolated and compared with 126 complete genome sequences from public databases. Whole-genome multi-locus sequence typing, resistance group analysis, genomic island prediction, and plasmid structural gene analysis were performed.

**Results:**

A total of 30 isolates were obtained from 230 samples, yielding a prevalence of 13.04%. All isolates exhibited multi-drug resistance, with 100% resistance to cotrimoxazole, erythromycin, penicillin G, chloramphenicol, ampicillin, and streptomycin. Among these, 15 isolates tested positive for the IncQ1α plasmid *repC* gene. The two most multi-drug-resistant and *repC*-positive strains, NO. 15 and 21, were sequenced to compare genomic features on Inc groups and ARGs with public data. Genome analysis revealed that the *repC* gene was primarily associated with IncQ1α, with structural genes from other F-type plasmids (*TraV*, *TraU*, *TraN*, *TraL*, *TraK*, *TraI*, *TraH*, *TraG*, *TraF*, *TraE*/*GumN*, and *TraA*) also present. Strain NO. 15 carried 33 ARGs, and strain NO. 21 carried 38 ARGs, conferring resistance to tetracyclines, fluoroquinolones, aminoglycosides, sulfonamides, peptides, chloramphenicol, cephalosporins, lincomycins, macrolides, and 2-aminopyrimidines.

**Conclusion:**

The *repC* gene is primarily associated with IncQ1α, with structural genes from other F-type plasmids. A comparison with 126 public genome datasets confirmed this association.

## 1 Introduction

The issue of antibiotic resistance (AMR) in *Enterobacteriaceae* bacteria is a serious and growing global public health problem. AMR increases mortality and prolongs disease progression. A meta-analysis reported that the mortality relative risk of carbapenem-resistant *Enterobacteriaceae* to carbapenem-susceptible ones is 2.14 (95%CI 1.85 to 2.48; I^2^ = 80.0%) (Zhou et al., 2021). Carbapenems are often used as “last-line agents” to defend against multidrug-resistant Gram-negative organisms. Tigecycline and polymyxin have been used to treat serious infections caused by carbapenemase-producing *Enterobacteriaceae*.

However, one member of the *Enterobacteriaceae* family*, Proteus mirabilis* (*P. mirabilis*), has been largely overlooked. *P. mirabilis* was reported as inherently resistant to polymyxin and tigecycline (Alqurashi et al., 2022). However, only a few articles have reported the risks associated with this inherent resistance, which can horizontally transferred to other *Enterobacteriaceae.*

*P. mirabilis* is a conditional pathogen primarily found in the intestines of animals, which was first identified and named by Hauser in 1885 (Drzewiecka, 2016). It belongs to the *Enterobacteriaceae* family within the genus *Proteus*, along with *P. penneri, P. vulgaris, P. myxofaciens, P. hauseris*, and three unnamed genomospecies (*Proteus* genomospecies 4, 5, and 6) (O’Hara et al., 2000). It is mainly found in the gastrointestinal tracts of humans and animals, and humans (Sanches et al., 2021), pigs (Qu et al., 2022), dogs (Kyung et al., 2024), chickens (Ramatla et al., 2024), fish (Anifowose et al., 2024), and other animals (Kang et al., 2021). If hosts become infected, it can lead to gastroenteritis (Ravindran et al., 2023), urinary tract infections (Chakkour et al., 2024), meningitis (Costa Filho et al., 2023), and other diseases. *P. mirabilis* has been used as an indicator of food and fecal contamination, which has led to an underestimation of its pathogenicity (Yu et al., 2021). In recent years, there have been increasing reports of *P. mirabilis* causing diseases in animals, which has had significant adverse effects on the livestock and poultry industry (Li et al., 2023). Due to the overuse of antibiotics, the losses caused by multidrug-resistant *P. mirabilis* are increasing. A prevalence of AMR *P. mirabilis* isolated from meat products in southern Brazil reported the high prevalence of multi-drug resistance (MDR) isolates in chicken (76.5%), which threatens the breeding industry seriously (Sanches et al., 2023).

The critical issue of antibiotic resistance extends beyond its direct impact to include the horizontal transfer of antibiotic resistance genes (ARGs) (Elhoshi et al., 2023). ARGs carried on plasmids can confer acquired antimicrobial resistance to recipient bacteria through mechanisms such as conjugation, transformation, and transduction (Mei et al., 2024). This HGT allows ARGs to spread rapidly among different bacterial species, exacerbating the problem of MDR (Darby et al., 2023). This spread of ARGs through plasmid-mediated HGT can rapidly transform sensitive bacteria into MDR. Typically, plasmids exhibiting MDR can be classified according to their incompatibility (Inc) because of the feature that plasmids cannot stably coexist with other plasmids within the same bacterial strains (Meinersmann, 2019). According to the Inc classification, plasmids could be divided into several groups (IncA, IncB, IncC, IncD, IncF, IncH, IncI, IncJ, IncK, IncL, IncM, IncN, IncO, IncQ, and IncP), with each group having distinct characteristics (Foley et al., 2021). The Inc plasmid typing method has become the most common approach for plasmid typing. Research on the correlation of incompatibility groups from different regions is a crucial tool for studying the genetic characteristics of plasmids.

*P. mirabilis* is widely distributed in the natural environment, with animal gastrointestinal tracts serving as essential vectors for the horizontal transmission of antibiotic resistance genes (ARGs). Due to its low pathogenicity, *P. mirabilis* has often been overlooked. However, it is inherently resistant to polymyxin tigecycline, raising concerns about its potential role in ARG transmission, which poses a significant public health threat.

This study aims to investigate the presence of Inc plasmid types in *P. mirabilis* isolated from pigs in Kunming, Yunnan. Specifically, 30 *P. mirabilis* strains were isolated from 230 pig fecal samples collected in Kunming between 2022 and 2023. These isolates were analyzed for AMR and the prevalence of Inc plasmid *rep* genes. Based on these results, two isolates with notable AMR profiles and mobile genetic elements were selected for whole-genome sequencing. Then, the complete genome data from this study were then compared with publicly available *P. mirabilis* data from NCBI. This study could provide a theoretical foundation for subsequent research on the transfer and dissemination of ARGs, as well as the mechanisms of drug resistance in *P. mirabilis*.

## 2 Materials and methods

### 2.1 Sample collection

From 2022 to 2023, a total of 230 fecal swab samples were collected from swine farms across various districts of Kunming. Specifically, 34 samples were collected from the Luquan district of Kunming in March 2022, 22 samples from the Jinning district in June 2022, 52 samples from the Yiliang district in August 2022, 33 samples from the Anning district in March 2023, 39 samples from Songming in June 2023, and 42 samples from the Xundian district in August 2023. The samples were placed in sterilized centrifuge tubes containing Brain Heart Infusion (BHI) agar, refrigerated, and promptly transported to the Yunnan Joint International R&D Center of Veterinary Public Health for bacterial culture. Initial characterization of the samples began on the day they arrived at the laboratory. This formula, n = (z)^2^p(1–p)/d^2^, was applied to calculate the required sample size. In the formula, the ‘n’ represents the required sample size; ‘z’ the level of confidence according to a standard normal distribution (for a level of confidence of 95%, z = 1.96); the ‘p’ the expected prevalence, and the ‘d’ allowable error (here it was set to be 5%). The expected prevalence was approximately 18.03% for *P. mirabilis*, according to the report by Rui et al. (2016).

### 2.2 *Proteus mirabilis* isolation and identification

The fecal swabs were inoculated in buffered peptone water (BPW, Huankai) and incubated in a constant temperature shaker at 37°C, 140 rpm/min for 12 h. Subsequently, 100 μL of the culture was transferred onto Xylose Lysine Deoxycholate (XLD) agar plates and incubated under the same conditions for an additional 12 h. Suspected colonies were selected, re-inoculated onto fresh XLD agar, and incubated at 37°C for 18 h. Finally, a single colony from suspected isolates was then purified by culturing on lysogeny broth (LB) medium. To confirm purity, all isolates were triple-passaged to obtain fresh colonies, followed by Gram staining and biochemical tests, including TSI (Triple Sugar Iron), urea, indole, phenylalanine deaminase, ornithine decarboxylase, and lactose utilization.

Preliminary identification was conducted by amplifying the sequences based on the GenBank-registered *ids* gene cluster of *P. mirabilis*. Bacteria with positive ids gene clusters were further analyzed by blasting the 16S rRNA genes. Primers are shown in Table 1.

**Table 1:** Primer information.

Primer	Sequences (5′-3′)	Length (bp)	Ref/accession
16SrRNA-F	5’-AGAGTTTGATCATGGCTCAG-3’	1,300	Tingyin Zhou (2009)
16SrRNA-R	5’-GTGTGACGGGCGGTGTGTAC-3’		
ids-F	5’-TTATACTCGCAACGGTGAAC-3’	829	NC_022000.1
ids-R	5’-AAATAACGGCTCTCGCTTAC-3’		

### 2.3 Antimicrobial susceptibility test

A total of 30 isolates were subjected to antimicrobial susceptibility testing using the disk diffusion method and minimum inhibitory concentration (MIC) assays. Both methods were conducted according to the guidelines of the American Clinical and Laboratory Standards Institute (CLSI). MIC results were interpreted according to the recommendations of the Clinical Laboratory Standard Institute guidelines (CLSI, 2018; Humphries et al., 2021). Isolates were classified as multidrug-resistant if they were resistant to three or more antimicrobial drugs in the panel (Dargatz et al., 2016). The 19 antibiotics from 8 categories included: aminoglycoside antibiotic have Neomycin (NEO, 30 μg) and Streptomycin (STR, 10 μg); Beta-lactam antibiotics have Cefaclor (CEC, 30 μg), Cefotaxime (CTX, 30 μg), Ceftriaxone (CRO, 30 μg), Cefepime (FEP, 30 μg), Penicillin (PEN,10 units) (for Staphylococcus spp.) or 1 unit (for Streptococcus spp.), Ampicillin (AMP, 10 μg), Imipenem (IPM, 10 μg); tetracycline class of antibiotics have Tigecycline (TGC, 15 μg) and Tetracycline (TC, 30 μg); Fluoroquinolones have Norfloxacin (NOR, 10 μg) and Ciprofloxacin (CIP, 5 μg); Amphenicols have Chloramphenicol (CHL, 30 μg); Macrolide antibiotics have Erythromycin (ERY, 15 μg); lincosamide antibiotics have Clindamycin (CLI, 2 μg); Sulfonamides and trimethoprim have Cotrimoxazole (SXT, 1.25/23.75 μg); While polymyxin antibiotics contain polymyxin E (colistin) and polymyxin B, they were tested using the MIC. The antimicrobial susceptibility results were evaluated based on the CLSI M100-S23 criteria.

### 2.4 Characterization of Inc plasmid

The *inc/rep* PCR method was used to detect replicons on reference plasmids (Carattoli et al., 2005). Eighteen pairs of primers were designed for 18 Inc plasmid genes, including *rep* and *par*. Primers are shown in Table 2. After the amplification reaction, the results were observed using 1% agarose gel electrophoresis.

**Table 2:** Primer information.

Primer	Target gene	Sequences (5′-3′)	Length (bp)	Ref/accession
IncQ1α-F	*repC*	AAGCCTAAGAACAAGCACAG	802	M28829.1
IncQ1α-R		CATAGCCGCACAAGGTATC		
IncG-F	*repA*	TGAGTTCATCAAGCCCAATC	872	KU578314
IncG-R		TGATAAGCGTGTCGTTCTTG		
IncL-F		ACAGAAGAGTAACCCGGAG	605	KM406489
IncL-R		ATTCTTTAGGGGACTGGCTT		
IncM-F		CGGCTCAGAATAGAATCAGG	612	KM406488
IncM-R		GTTCCCTTCGCTGTCTTTTT		
IncR-F	*repB*	GCGTTCTCTGGTTATGTCTT	529	KY296104
IncR-R		GCAGGATCAAGGAAAGATCG		
IncU-F	*repA-repB*	AACGTCAATCCTCTTTCCCT	940	CR376602
IncU-R		TCGTTTTTGGGCGTGTATAG		
IncN-F	*repA*	GCGAAGATGATGATGAGATGGC	306	AY046276
IncN-R		GGAGCGAGTAGGTGGTGAAC		
IncA/C-F	*repA*	GAACGCCAGGTGCTATG	415	X141473
IncA/C-R		CTCTGTCTGCTGCTTACG		
IncW-F	*repA*	TGGCTTAGTCGGCTACAT	493	BR000038
IncW-R		TCGGATAGGAATCGGTGAG		
IncP-F	*repA*	GGCGAAGTAGTCGAACAT	598	L27758
IncP-R		GAAGCAGCAGATCAAGGA		
IncX1-F	Unspecified target gene	GCTTAGACTTTGTTTTATCGTT	461	Qu et al. (2022)
IncX1-R		TAATGATCCTCAGCATGTGAT		
IncX2-F		GCGAAGAAATCAAAGAAGCTA	678	
IncX2-R		TGTTGAATGCCGTTCTTGTCCAG		
IncX3-F		GTTTTCTCCACGCCCTTGTTCA	351	
IncX3-R		CTTTGTGCTTGGCTATCATAA		
IncX4-F		AGCAAACAGGGAAAGGAGAAGACT	569	
IncX4-R		TACCCCAAATCGTAACCTG		
IncHI1-F	*ParA-ParB*	GGAGCGATGGATTACTTCAGTAC	471	Kyung et al. (2024)
IncHI1-R		TGCCGTTTCACCTCGTGAGTA		
IncI1-F	*RNAI*	CGAAAGCCGGACGGCAGAA	139	
IncI1-R		TCGTCGTTCCGCCAAGTTCGT		
IncFIB-F	*repA*	GGAGTTCTGACACACGATTTTCTG	702	
IncFIB-R		CTCCCGTCGCTTCAGGGCATT		
IncT-F	*repA*	TTGGCCTGTTTGTGCCTAAACCAT	750	
IncT-R		CGTTGATTACACTTAGCTTTGGAC		

### 2.5 Plasmid conjugation test

The characteristic gene-positive isolates from the 30 strains were used as donor strains in a conjugation test. The recipient strain was an engineered *Escherichia coli* DH5α. Donor and recipient strains were mixed in a 4:1 ratio and inoculated into LB medium, followed by incubation at 37°C for 4 h. Subsequently, 100 μL of the donor, recipient, and conjugated cultures were plated on MacConkey agar containing antibiotics to which all strains were resistant. The plates were then incubated at 37°C for 24 h.

### 2.6 Whole genome sequence and preparation of public complete genome data

Isolates exhibiting severe MDR and containing transfer elements were selected for whole-genome sequencing. DNA was extracted using SDS combined with a purification column. Then, the genome was quality-checked. Qualified DNA was randomly fragmented using Covaris. The ONT SQK-LSK109 and EXP-NBD104/114 kits (Oxford Nanopore Technologies^1^) were used for online library construction: the qualified DNA samples were purified using magnetic beads to select DNA fragments with an average size of 200–400 bp.

The 1 μL sample was taken for Qubit quantification, damage repair, end-repair, barcode labeling, pooling library preparation, and sequencing junction ligation. Their whole genomes were sequenced using Illumina MiSeq and Oxford Nanopore MinION platforms. The genomes of this study were assembled using the NECAT software, which used long-read data from the Nanopore. Subsequently, the final high-quality assembly was achieved using Pilon software (v.1.22) to correct the Illumina MiSeq sequencing data based on NGS reads. Multiple assembly versions were mirrored to create the final map.

This study employed the complete level whole genome data from both self-isolated and sequenced strains, as well as 126 publicly available strains. The public complete genome data for 126 strains of *P. mirabilis* were downloaded from the NCBI database on March 17, 2024. The whole genome data of the self-isolated strains were annotated using the GO, KEGG, and RAST^2^ databases.

### 2.7 Whole genome multi-locus sequence typing (wgMLST)

PubMLST^3^ was used to analyze the genomic relationship of selected *P. mirabilis* and the published strains based on their location and sampling time. The 16 representative strains from various years and regions were selected. Gene loci were plotted using Entro-wgMLST v1.0. The phylogenetic trees were graphed using ggplot2 in R.

### 2.8 Resistome analysis

The resistome (the entire set of ARGs) of *P. mirabilis* was analyzed using the Comprehensive Antibiotic Resistance Database (CARD^4^).

The expression of resistance gene types and the phylogenetic analysis of ARGs based on sequence site information across the whole genome datasets were conducted.

### 2.9 Genome island prediction and plasmid structure gene analysis

IslandPath 1.0.6 software was employed to predict the genome islands and potential horizontal gene transfers using the sequence composition prediction method. The expression of genome island-related genes in the whole-genome-sequenced strains in this study was analyzed. SnapGene Viewer 6.1.2 software was used for the gene island visualization.

The annotation results of these genomes were examined to determine the expression of transfer-related genes. The proportion of Inc plasmid structure genes in *P. mirabilis* was determined. The presence or absence of repA and repC, along with their sequence site information, was analyzed for evolutionary relationships.

## 3 Results

### 3.1 Isolation and prevalence of *P. mirabilis*

**Figure 1:**
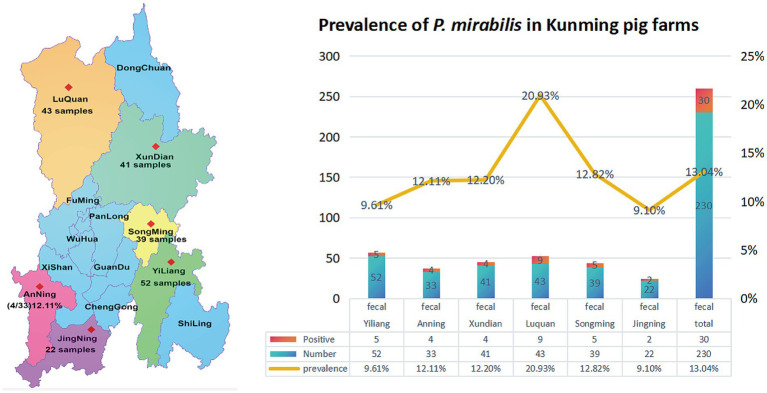
Prevalence of *P. mirabilis* in Kunming, Yunnan, China.

The *Ids* gene was amplified by PCR. The positive *P. mirabilis* product was 829 bp. The *16S rRNA* gene PCR amplification produced a band at 1,300 bp, which was sequenced and compared using NCBI’s BLAST software.^5^ It revealed that all isolates had more than 98% nucleic acid sequence homology with *P. mirabilis*. Ultimately, 30 isolates were obtained from 230 samples, resulting in a prevalence of 13.04% (see Figure 1).

### 3.2 Antimicrobial susceptibility test results

**Figure 2:**
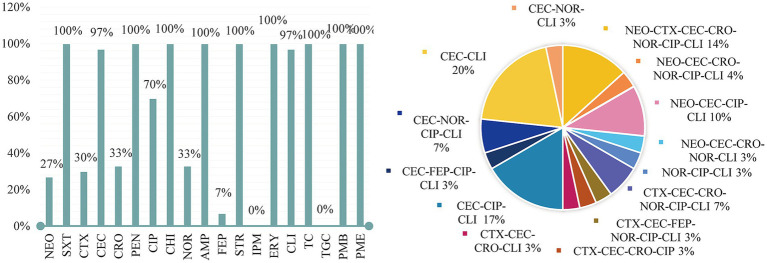
Antimicrobial susceptibility results.

The results were evaluated according to the CLSI M100-S23 criteria by the diameter of the inhibitory zone. The summary results are presented in Figure 2. Overall, among all *P. mirabilis* isolates (n = 30), the highest frequency of resistance was observed against SXT, ERY, PEN, AMP, CHI, STR, TC, and TGC (100%), followed by CEC (97%), CLI (97%), and CIP (70%). All 30 isolates exhibited MDR. Specifically, 4 isolates were resistant to 16 drugs is strain 16, 21, 22, 25 (AMP-ERY-PEN-SXT-STR-PMB-PMC-TC-CEC-CLI-NEO-CET-CRO-NOR-CIP-CHI), 3 isolates to 15 drugs is strain 13, 17 (AMP-ERY-PEN-SXT-STR-PMB-PMC-TC-CEC-CLI-CET-CRO-NOR-CIP-CHI), and strain 15 (AMP-ERY-PEN-SXT-STR-PMB-PMC-TC-CEC-CLI-NEO-CRO-NOR-CIP-CHI), 1 isolate to 14 drugs is strain 8 (AMP-ERY-PEN-SXT-STR-PMB-PMC-TC-CEC-CLI-CET-FEP-CIP-CHI), 8 isolates to 13 drugs is strain 3, 23, 24 (AMP-ERY-PEN-SXT-STR-PMB-PMC-TC-CLI-NEO-CET-CIP-CHI); and strain 7, 9 (AMP-ERY-PEN-SXT-STR-PMB-PMC-TC-CEC-CLI-FEP-CIP- CHI); and strain 7 (AMP-ERY-PEN-SXT-STR-PMB-PMC-TC-CEC-CLI-NOR-CIP-CHI); and strain 10 (AMP-ERY-PEN-SXT-STR-PMB-PMC-TC-CEC-CET-CRO-CIP-CHI); and strain 26 (AMP-ERY-PEN-SXT-STR-PMB-PMC-TC-CEC-CLI-NEO-CRO-CHI); 8 isolates to 12 drugs is strain 2, 4, 5, 19, 20 (AMP-ERY-PEN-SXT-STR-PMB-PMC-TC-CEC-CLI-CIP-CHI); and strain 1 (AMP-ERY-PEN-SXT-STR-PMB-PMC-TC-CLI-NOR-CIP-CHI); and strain 14 (AMP-ERY-PEN-SXT-STR-PMB-PMC-TC-CEC-CLI-CET-CHI); and strain 30 (AMP-ERY-PEN-SXT-STR-PMB-PMC-TC-CEC-CLI-NOR-CHI), 6 isolates to 11 drugs is strain 11, 12, 18, 27, 28, 29 (AMP-ERY-PEN-SXT-STR-PMB-PMC-TC-CEC-CLI-CHI).

For antimicrobial resistance, 14 resistance patterns were identified, with the most common being the 10-resistance pattern (AMP-ERY-PEN-SXT-STR-PMB-PMC-TC-CEC-CLI), accounting for 20%, followed by 12-resistance (17%), 15-resistance (14%). The MDR rate (resistant to over nine types of antimicrobials) reached 100%, as shown in Figure 2. According to Magiorakos et al. (2012), the isolated strains were classified into MDR, XDR, and PDR categories, which can be viewed in the attached table.

### 3.3 Inc plasmid characterization gene results

**Figure 3:**
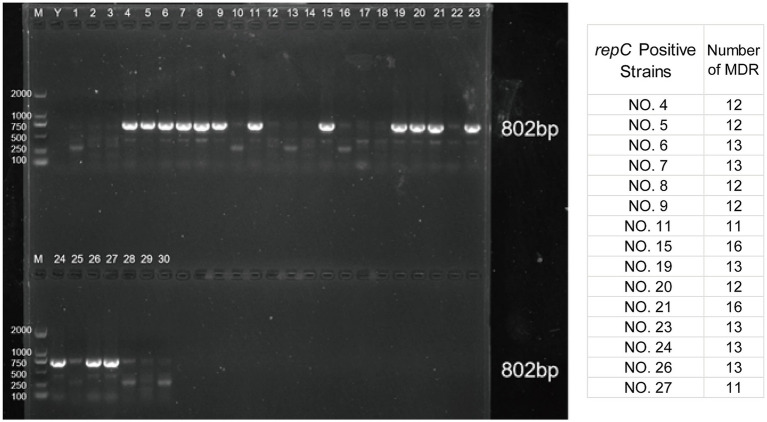
PCR amplification results of IncQ1𝛼 plasmid *repC* gene. M lane is DL2000 Maker, Y is a negative sample, and lanes 1–30 are samples.

The results indicated that 15 isolates were positive for the IncQ1α plasmid *repC* gene, specifically isolates 4–9, 11, 15, 19–21, 23, 24, 26, and 27, with a detection rate of 50%. The *repC* gene amplification results for IncQ1α were shown in Figure 3, with amplified bands appearing at 802 bp, consistent with the expected results. The remaining isolates were negative for the Inc marker genes. According to the antimicrobial susceptibility results, two multi-drug-resistant and growth-dominant strains were selected for subsequent experiments. Considering the number of multi-drug-resistant in the *repC*-positive isolates, it can be inferred that NO. 15 and NO. 21 were suitable subjects for further research.

### 3.4 Conjugation assay results

IncQ plasmid-positive isolates formed colorless and transparent colonies on MacConkey’s medium supplemented with 10 μg/mL ampicillin, 30 μg/mL tetracycline, 5 μg/mL ciprofloxacin, and 15 μg/mL erythromycin. The control strain, *E. coli* DH5α, did not grow under these conditions. The control strain, *E. coli* DH5α, did not grow under these conditions. No transfer events were observed in any of the conjugation experiments.

### 3.5 Whole genome sequence of two *P. mirabilis*

Isolates NO. 15 and NO. 21 were both 16-drug-resistant strains and tested positive for the IncQα1 plasmid *repC* gene.

**Figure 4:**
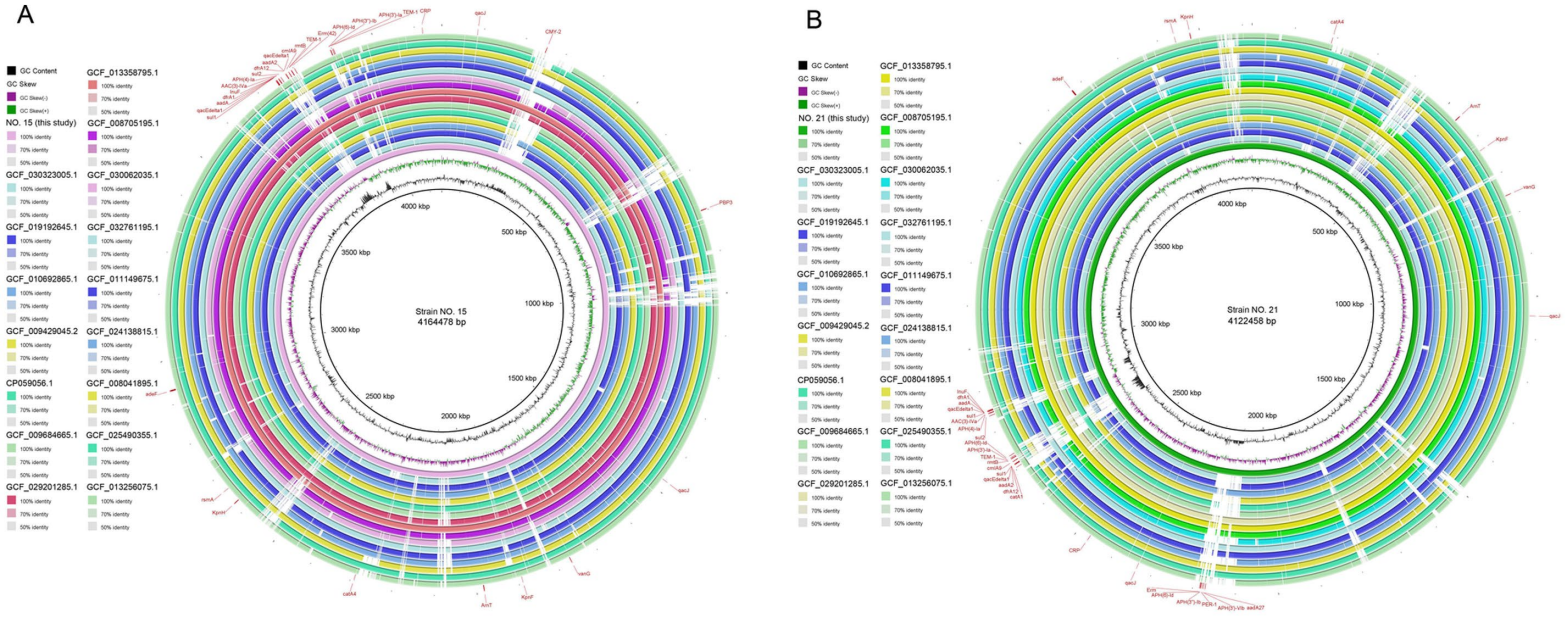
Parts A and B are a whole genome mapping of strains 15 and 21. From the outer to the inner circle: Target strain genome location coordinates; positive chain restriction-modification enzyme coding genes (color corresponding to COG classification); negative chain restriction-modification enzyme coding genes (color corresponding to COG classification); rRNA and tRNA distribution; Genome GC skew value (the specific algorithm is G−C/G + C). The inward purple part indicates that the content of G is lower than that of C in this region, and the outward pink part is the opposite: genomic GC content (the outward green part indicates that the GC content of this region is higher than the average GC content of the whole genome, and the inward blue part is the opposite, and the higher the peak value, the greater the difference from the average GC content).

The assembled genome sequences and functional annotation were mapped using the BLAST Ring Image Generator (BRIG 0.95). The whole genome landscapes of strains 15 and 21 are shown in Figure 4.

### 3.6 The wgMLST results

**Figure 5:**
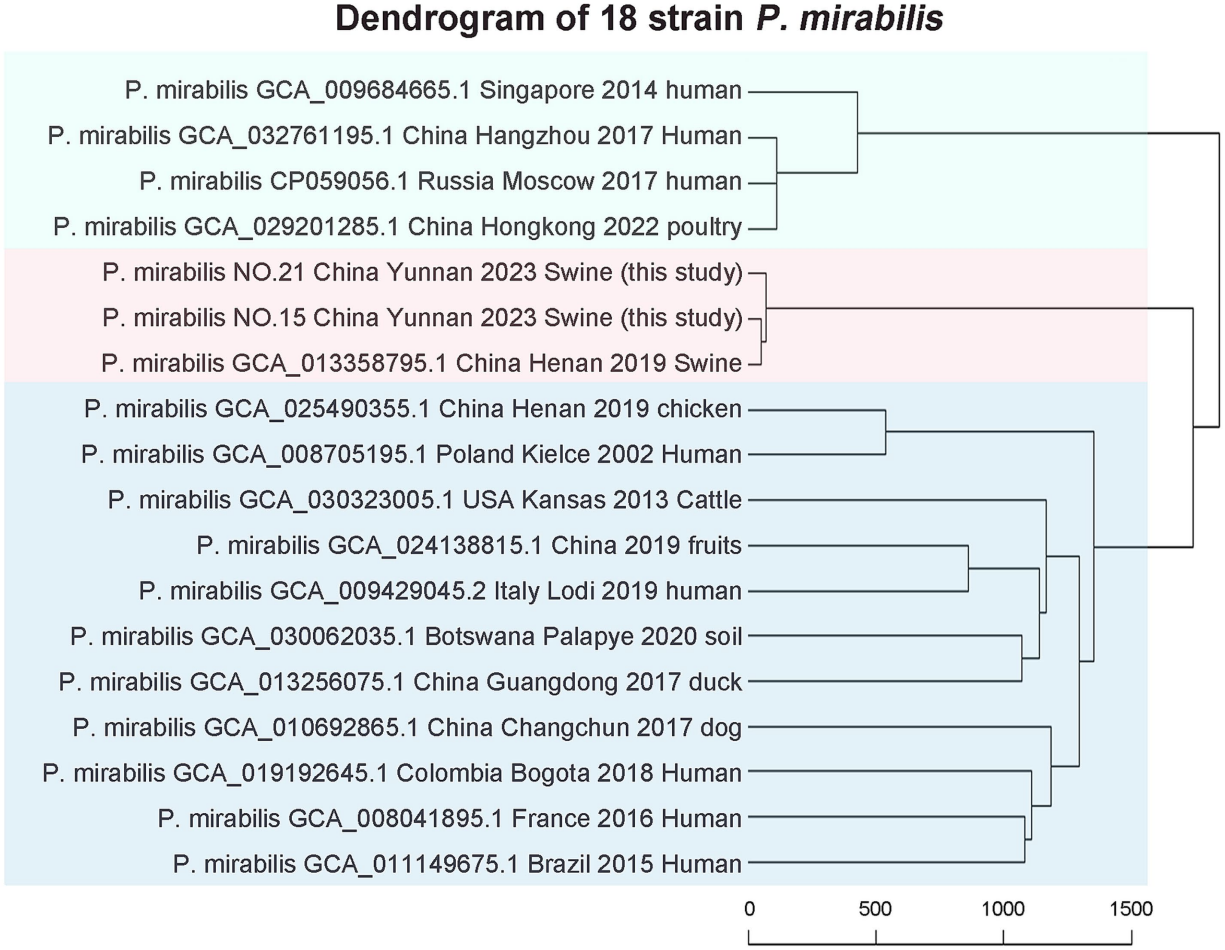
Phylogeny of the whole genomes of strain 15 and 21.

This study analyzed two strains of *P. mirabilis* and 16 strains from diverse global sources using whole-genome phylogenetic tree analysis. The results revealed three major strain groups. Interestingly, no correlations were observed among *P. mirabilis* strains from human, food, or animal origins. This indicates a close relationship between human-derived *P. mirabilis* and those found in food, animals, and pets. The two strains studied closely resembled porcine-derived *P. mirabilis,* reported in 2019 in Henan, China (NCBI genome assembly number GCA_013358795.1) (see Figure 5).

### 3.7 Resistome results

The sequences of strains NO. 15 and 21 were compared with the CARD databases using the Resistance Gene Identifier (RGI). Resistance gene annotation and statistical analysis were conducted for sequences with an identity greater than 95%. The antimicrobial resistance gene information for strains 15 and 21 is presented in Table 3.

**Table 3:** Information on resistance genes of strains 15 and 21.

Genes	Resistance profile	Mechanism	Strain15	Strain21
*adeF*	Tetracyclines, fluoroquinolones	Efflux pump	+	+
*rsmA*	Diaminopyrimidines, chloramphenicol, fluoroquinolones	Efflux pump	+	+
*kpnH*	Polypeptides, aminoglycosides, macrolides, cephalosporins, carbapenems, fluoroquinolones	Efflux pump	+	+
*catA4*	Chloramphenicol	Inactivation	+	+
*arnT*	Polypeptides	Target change	+	+
*kpnF*	Polypeptides, cephalosporins, rifamycin, disinfectants and antiseptics, tetracycline, aminoglycosides, macrolides	Efflux pump	+	+
*vanG*	Vancomycin	Target change	+	+
*qacJ*	Quaternary	Efflux pump	+	+
*erm (42)*	Macrolides, streptogramin, lincosamides	Target change	+	+
*erm*	Macrolides, streptogramin, lincosamides	Target change	−	+
*aph(6)-Id*	Aminoglycosides	Inactivation	+	+
*aph(3″)-Ib*	Aminoglycosides	Inactivation	+	+
*per-1*	Cephalosporins, carbapenems	Inactivation	+	−
*aph(3′)-VIb*	Aminoglycosides	Inactivation	+	−
*aadA27*	Aminoglycosides	Inactivation	+	+
*CRP*	Macrolides, fluoroquinolones	Efflux pump	+	+
*tet(G)*	Tetracyclines	Efflux pump	+	−
*dfrA1*	Diaminopyrimidines	Target replacement	+	−
*ANT(3″)-Ia*	Aminoglycosides	Inactivation	+	−
*qacE delta1*	Disinfectant and antiseptic	Efflux pump	+	+
*sul1*	Sulfonamides	Target replacement	+	+
*AAC(3)-IVa*	Aminoglycosides	Inactivation	+	+
*aph(4)-Ia*	Aminoglycosides	Inactivation	+	+
*sul2*	Sulfonamides	Target change	+	+
*aph(3′)-Ia*	Aminoglycosides	Inactivation	+	−
*tEM-1*	Penicillin, first-generation cephalosporins	Inactivation	+	+
*rmtB*	Penicillin, first-generation cephalosporins	Target change	+	+
*cmLA9*	Chloramphenicol	Efflux pump	+	−
	Phenols	Efflux pump	−	+
*aadA2*	Aminoglycosides	Inactivation	+	+
*dfrA12*	Diaminopyrimidines	Target replacement	+	+
*catA1*	Chloramphenicol	Inactivation	+	+
*lnuF*	Lincosamides	Inactivation	+	+
*qnrA1*	Fluoroquinolones	Target protection	+	+
*aadA1*	Aminoglycosides	Inactivation	−	+
*catA1*	Chloramphenicol	Inactivation	−	+
*aph(3″)-Ia*	Aminoglycosides	Inactivation	−	+
*CMY-2*	Cephalosporins, carbapenems	Inactivation	−	+
*PBP3*	Cephalosporins	Target change	−	+
*Tet(J)*	Tetracyclines	Efflux pump	−	+
*floR*	Chloramphenicol	Efflux pump	−	+
*aadA27*	Aminoglycosides	Inactivation	−	+

Strain NO. 15 carried 33 ARGs, with *qacJ* present in two subtypes. Ten were efflux pump genes, 14 genes in synthesizing antibiotic-inactivating enzymes, five genes in altering therapeutic targets, and three genes in substituting therapeutic targets. One gene was involved in protecting the therapeutic target. These genes confer resistance to a wide range of antibiotics, such as tetracyclines, fluoroquinolones, aminoglycosides, sulfonamides, polypeptides, chloramphenicols, cephalosporins, lincosamides, macrolides, and 2-aminopyrimidine.

Strain NO. 21 carried 38 ARGs, including two subtypes of *qacJ*, *sul1*, *catA1*, and *APH (6)-Id* genes. Among the ARGs, 11 were involved in efflux pumps, 16 in synthesizing antibiotic-inactivating enzymes, 7 in target alteration, 3 in target substitution, and 1 in target protection. These genes mediate resistance to tetracyclines, fluoroquinolones, aminoglycosides, sulfonamides, polypeptides, chloramphenicols, cephalosporins, lincosamides, macrolides, and 2-aminopyrimidines.

**Figure 6:**
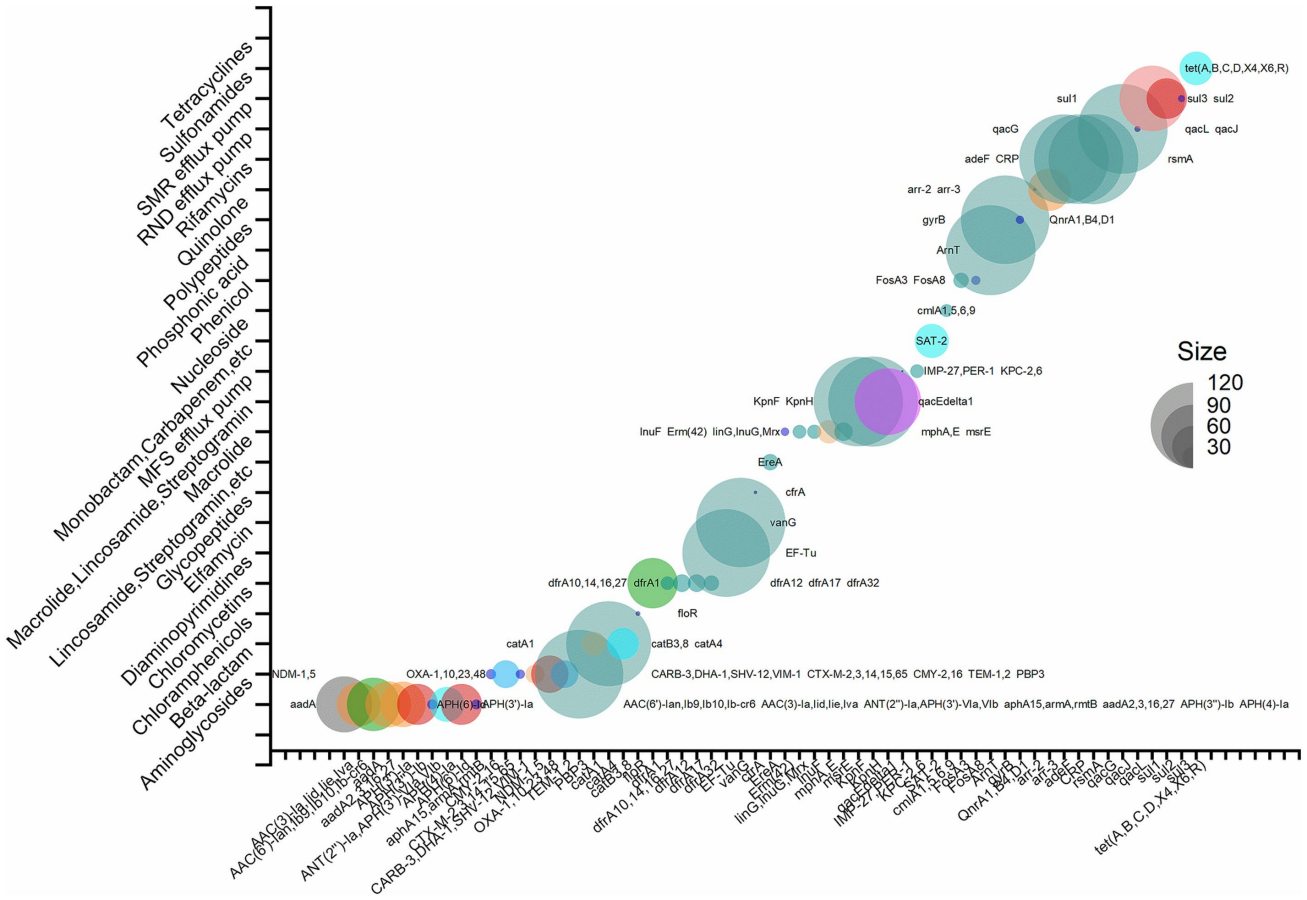
Resistome bubble plots of 128 strains of *P. mirabilis* (126 strains with publicly available data and two strains in this study).

To identify the most prevalent resistance genes in *P. mirabilis*, a meta-resistome analysis based on 128 strains (the 126 publicly available data and two strains of this study) of *P. mirabilis* was performed. According to the annotation results, 105 antimicrobial resistance genes were identified. These genes resisted various antibiotics, including aminoglycosides, beta-lactams, cephalosporins, chloramphenicols, chloromycetin, diaminopyrimidines, elfamycin, glycopeptides, oxazolidinone, phenicol, pleuromutilin, macrolide, lincosamide, streptogramin, monobactam, carbapenem, cephalosporin, penam, nucleoside, phosphonic acid, polypeptides, quinolone, rifamycins, and tetracyclines. The MFS, SMR, and RND efflux pumps were also discovered. There were 12 genes expressed in 90% of the strains. They were cephalosporins ARG *PBP3*; chloramphenicols ARG *catA4*; elfamycin ARG *EF-Tu*; glycopeptides ARG *vanG*; polypeptides ARG *ArnT*; quinolone ARG *gyrB*; MFS efflux pump-related genes *KpnE*, *KpnF*; the RND efflux pump-related genes *adeF*, *rsmA*, and *CRP*, the SMR efflux pump-related gene *qacJ.* Notably, the polypeptide gene *ArnT* was present in 99.2% of *P. mirabilis strains*. Alternatively, it might elucidate the principal cause of its intrinsic resistance to polymyxin. Details are shown in Figure 6. Supplementary material presents the phylogenetic relationships of the primary resistance genes (*vanG*, *rsmA*, *PBP3*, *KpnH*, *KpnF*, *gyrB*, *EF-TU*, *CRP*, *catA4*, *ArnT*, and *adeF*), including those from 2 strains in this experiment and 16 representative various region strains from public data.

### 3.8 Results of genome island prediction and plasmid structure genes

#### 3.8.1 Genome island prediction results

Strain No. 15 contained nine genome islands, totaling 350,110 bp and an average length of 38,901 bp. Strain No. 21 contained eight genome islands, with a total length of 343,701 bp and an average length of 42,962 bp, as shown in Table 4. Strain NO. 15 contains 20 ARGs across nine genome islands, representing 60.61% (20/33) of its total ARGs. Similarly, NO. 21 has 27 ARGs distributed over eight genome islands, accounting for 71.05% (27/38) of its total ARGs.

**Table 4:** Statistical results of genome island prediction for strains NO. 15 and 21.

Strain NO. 15	Length (bp)	Gene number	ARGs number	Strain NO. 21	Length (bp)	Gene number	ARGs number
Island1	105,862	100	1	Island1	10,109	13	0
Island2	36,524	62	0	Island2	54,113	73	0
Island3	11,066	11	0	Island3	4,165	8	0
Island4	26,620	31	0	Island4	96,587	93	8
Island5	9,179	12	0	Island5	22,029	19	0
Island6	34,425	48	0	Island6	79,593	91	12
Island7	73,586	80	14	Island7	42,632	43	7
Island8	31,533	34	5	Island8	34,473	52	0
Island9	21,315	19	0				

#### 3.8.2 The expression of genome island-related genes

**Figure 7:**
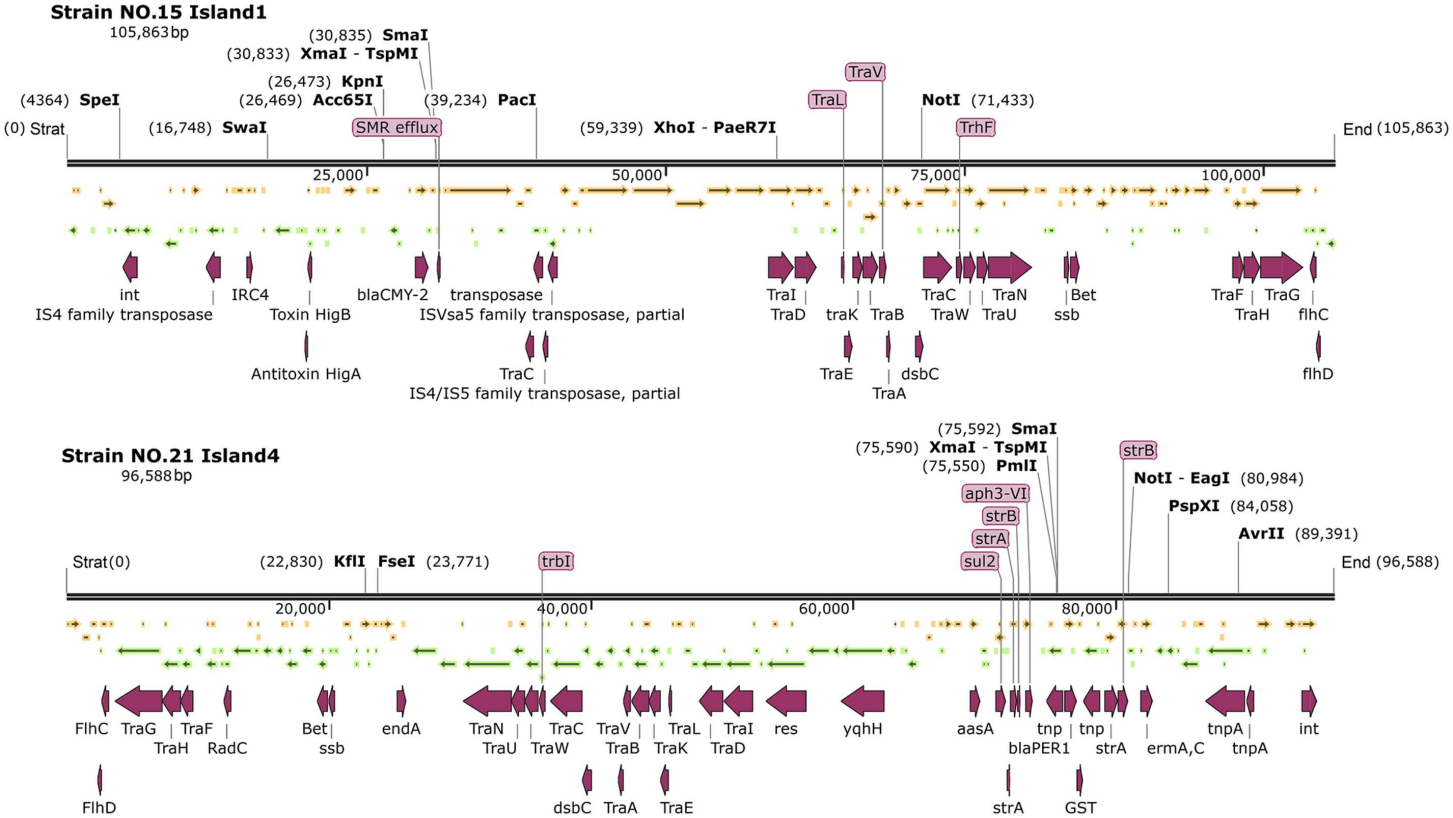
Genetic maps of strain NO. 15 island 1 and NO.21 island 4.

In these two *P. mirabilis* strains, several typical F-type plasmid conjugation transfer region structure genes of the T4SS secretion system, such as *traF*, *traH*, *traG*, *traN*, *traU*, *traW*, *traC*, *traV*, *traB*, *traA*, *traE*, *traD*, and *traI,* were present in Island 1 of strain No. 15 and Island 4 of strain NO. 21. These structural genes were predominantly clustered together. Both plasmids contain transposons such as the integrator int., the *flhCD* promoters, and *tnpA* (see Figure 7).

**Figure 8:**
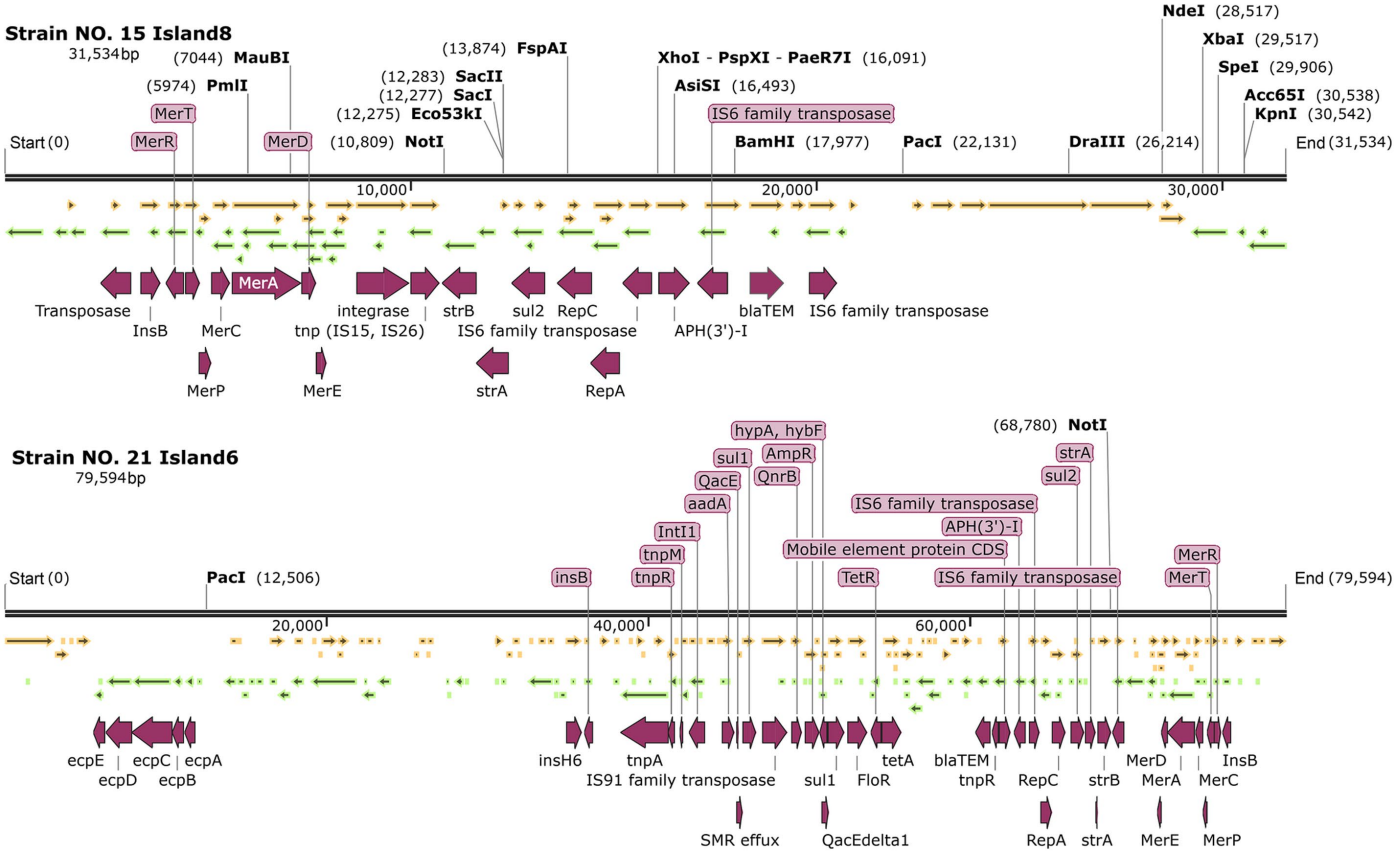
Genetic maps of strain NO. 15 island 8 and NO.21 island 6.

In the islands containing the *repC* gene of IncQ1α, both NO. 15 island 8 and NO.21 island 6 harbor the *repC* and *repA* genes. The related ARGs included the mercury resistance genes, bla-*TEM*, *strA*, *strB*, *sul* genes, and various transposons such as *tnp* (see Figure 8).

#### 3.8.3 The proportion of Inc plasmid structure genes

**Figure 9:**
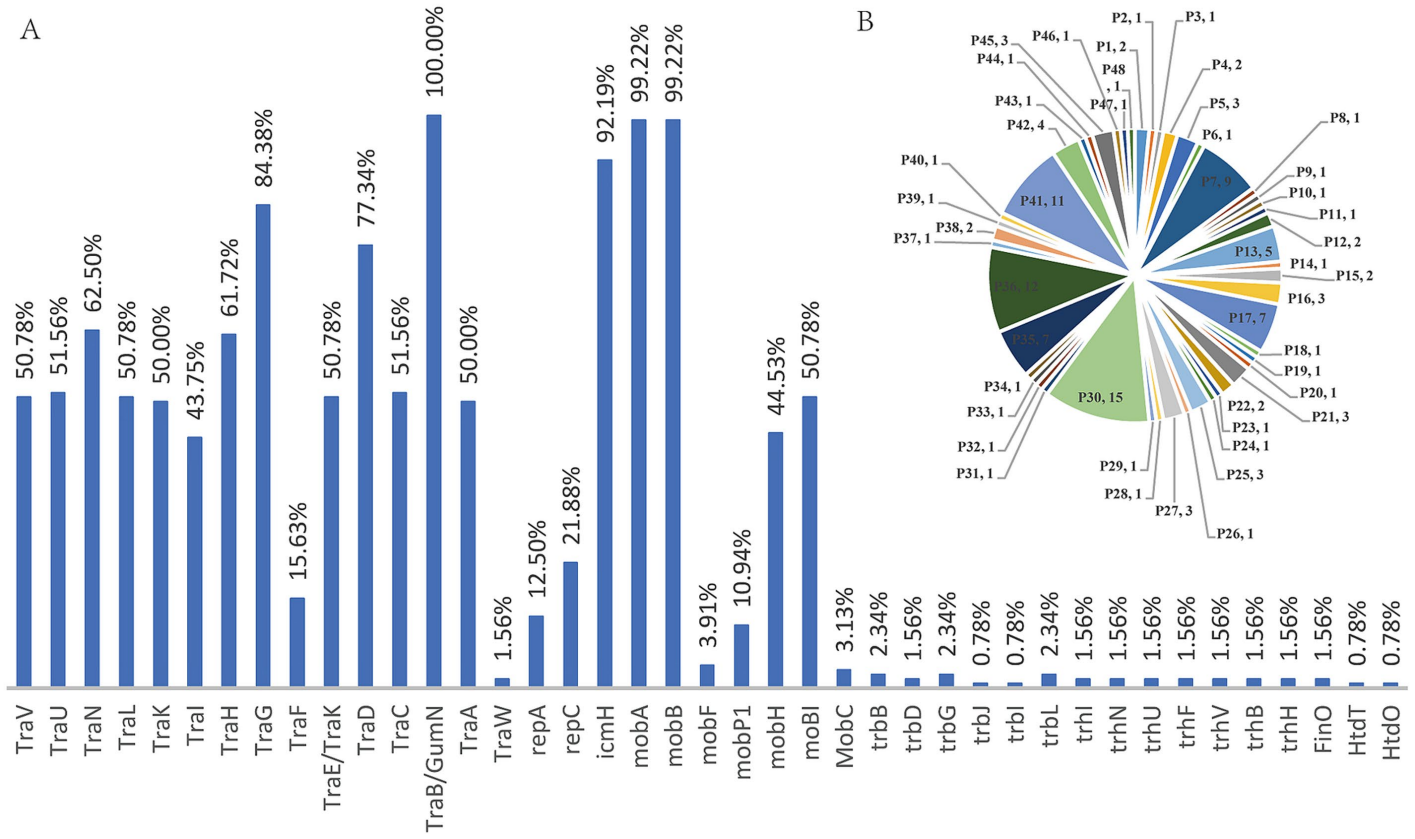
Presence of type-F plasmid structural genes among 128 genome data.

The annotation results of 128 genomes were analyzed to determine the proportion and composition of each structural gene. The proportions were as follows: *TraV* (50.78%), *TraU* (51.56%), *TraN* (62.50%), *TraL* (50.78%), *TraK* (50.00%), *TraI* (43.75%), *TraH* (61.72%), *TraG* (84.38%), *TraF* (15.63%), *TraE*/*GumN* (100%), *TraA* (50.00%), and *TraW* (1.56%). Additionally, *icmH* was 92.19%, *mobA* was 99.22%, *mobB* was 99.22%, *mobP1* was 10.94%, *mobH* was 44.53%, and *moBI* was 50.78%. Other genes had proportions less than 5%. Classification of their expression patterns revealed that they all contained type-F plasmid structural genes; the patterns included 48 distinct types among the 128 bacterial strains, indicating no correlation between them (see Figure 9).

#### 3.8.4 The presence of *repA* and *repC* genes with their evolutionary relationship results

From 128 whole genome datasets, 26 *repC* genes were identified. Among these, the *repC* genes of GCF013357505 and GCF011383025 were incomplete, resulting in 24 complete datasets. In this study, 15 *repC* gene sequences were obtained through sequencing. Notably, all 41 *repC* genes belong to the IncQ1 group. Combining these 15 sequences with the 24 publicly available ones, an evolutionary tree was constructed using the maximum likelihood method using MEGA 5.0 software. These *repC* genes were sorted into three groups. The overall mean genetic distance of the *repC* genes (15 sequences in this study and the 24 publicly available ones) was 0.006 ± 0.001.

**Figure 10:**
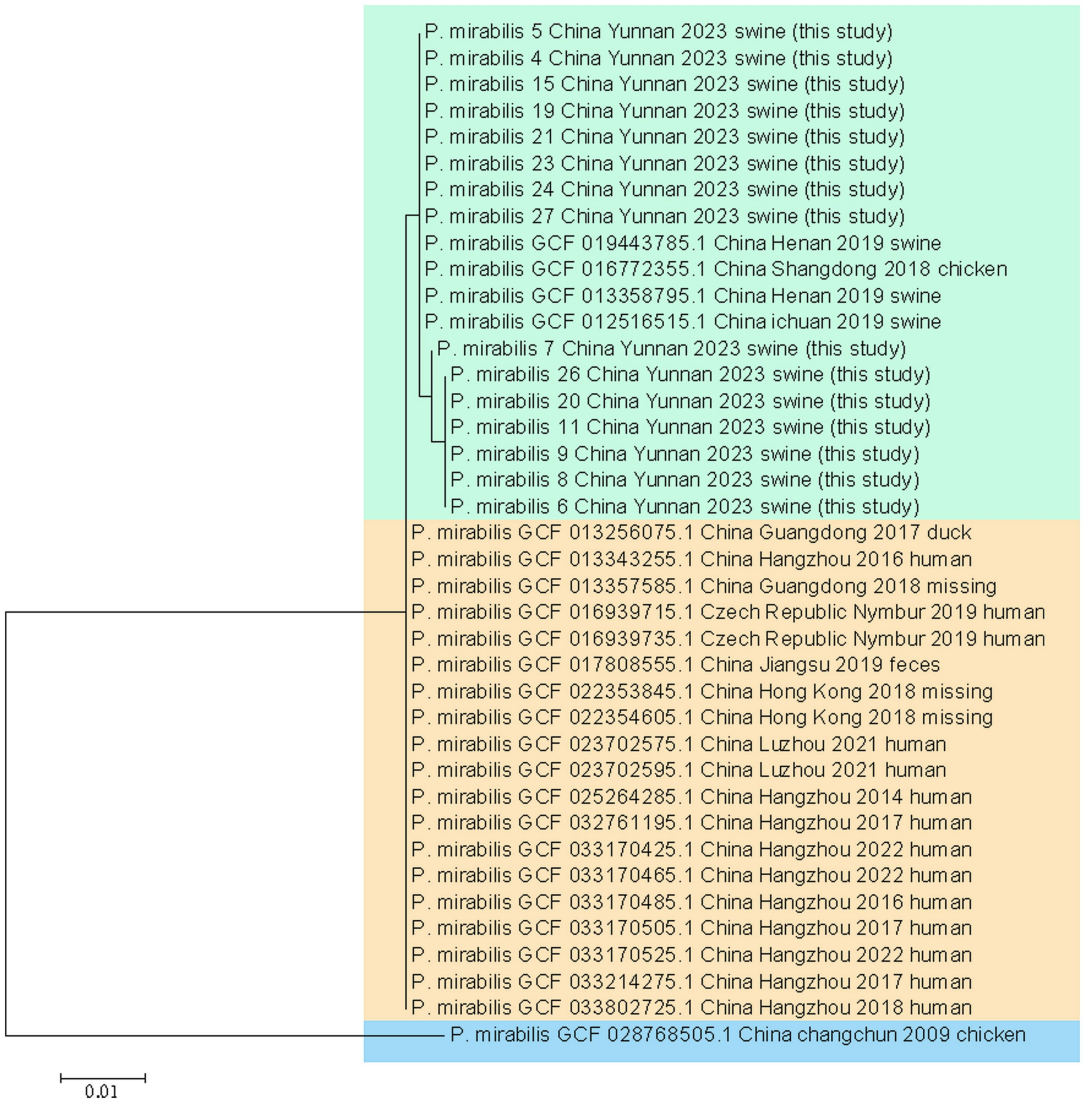
Phylogenetic tree of the IncQ1α plasmid *repC* positive strains.

A total of 14 *repA* genes were annotated across 126 public data strains. Among these, the *repA* of GCF002197405.1 was identified as IncN and GCF0230895.1 as IncFII. The remaining 11 strains (GCF_014843115.1, GCF_015169015.1, GCF_018972025.2, GCF_025490355.1, GCF_026016045.1, GCF_026016105.1, GCF_026016125.1, GCF_026016145.1, GCF_033170445.1, GCF_033215415.1, and GCF_033439945.1) contained incomplete *repA* genes with consistent sequence expression. These genes are closely related to sequences such as the *Vibrio alginolyticus* strain C1579 plasmid pC1579 and *Shewanella* aestuarii strain PN3F2 plasmid pPN3F2_1, among others. Additionally, the *repA* strain GCF002180235.1 is closely related to the *Escherichia coli* strain BK31611 plasmid pBK31611. The two *repA* strains analyzed in this study show a close plasmid relationship with RSF1010 (IncQ-1α plasmid), pO26-CRL-125 (IncK2 plasmid), and p0716-KPC (IncFII plasmid) (see Figure 10).

## 4 Discussion

*Proteus mirabilis* is a conditionally pathogenic bacterium that is commonly found in the natural environment and animals (Girlich et al., 2020). It was reported to be inherently resistant to polymyxin and tigecycline. Horizontal transfer of resistance genes can transform drug-sensitive bacteria into multidrug-resistant strains rapidly. The inherent resistance of *P. mirabilis* poses significant risks if such a transfer occurs. However, this topic has been largely overlooked due to the severe neglect of its conditional pathogenicity. This study aims to investigate the presence of Inc plasmid types in pig-derived *P. mirabilis* isolated from Kunming, Yunnan. The 30 strain of *P. mirabilis* were successfully isolated from 230 pig fecal samples in Kunming, with a prevalence of 13.04% (30/230). This was consistent with that of Chinnam et al. (2021), who found 23 and 26 strains in 160 pork samples and 163 rectal swabs of normal pigs in Andhra Pradesh, India, with prevalences of 14.38 and 15.95%, respectively.

The wgMLST results included 16 representative strains from various years and regions, along with two strains from this study. They exhibited no relationship among *P. mirabilis* strains from human, food, or animal origins, meaning a close genomic relationship between human-derived *P. mirabilis* and the strains found in food, animals, and pets. The two strains studied closely resembled porcine-derived *P. mirabilis, which was* reported in 2019 in Henan, China (NCBI genome assembly number GCA_013358795.1).

The antimicrobial susceptibility results exhibited a serious AMR in the pig-derived *P. mirabilis* in Kunming, Yunnan. All the strains were MDR, with 100% resistance to cotrimoxazole, erythromycin, penicillin G, chloramphenicol, ampicillin, and streptomycin. *P. mirabilis* was naturally resistant to tetracycline and polymyxin with a resistance rate of 100% (Alqurashi et al., 2022), which is consistent with the current experimental results. The MDR rate in this study reached 100%, which was higher than the 76.7% reported in Northeast China (Sun et al., 2020) and the 78.13% reported in Brazil (Sanches et al., 2019) of the *P. mirabilis* isolates from chickens, which fully reflects the MDR of *P. mirabilis*. These results highlighted a significant drug resistance issue in Kunming pig-derived *P. mirabilis*, warranting attention. It may serve as a reservoir of resistance genes within the gut microbiota.

The resistome results of the two whole-genome-sequenced strains revealed that strain NO. 15 harbored a total of 33 ARGs, while strain NO. 21 carried 38 ARGs. These genes confer resistance to a wide range of antibiotics, including tetracyclines, fluoroquinolones, aminoglycosides, sulfonamides, polypeptides, chloramphenicols, cephalosporins, lincosamides, macrolides, and 2-aminopyrimidine. The resistome results from the genome of 126 strains of public data and two strains in this study revealed that 12 genes were expressed in 90% of the strains, including Cephalosporins ARG *PBP3*, Chloramphenicols ARG *catA4*, Elfamycin ARG *EF-Tu*, Glycopeptides ARG *vanG*, Polypeptides ARG *ArnT*, Quinolone ARG *gyrB*, MFS efflux pump-related genes *KpnE* and *KpnF*, RND efflux pump-related genes *adeF*, *rsmA*, and *CRP*, and the SMR efflux pump-related gene *qacJ*. Notably, the polypeptide gene *ArnT* was present in 99.2% of *P. mirabilis* strains, potentially explaining its intrinsic resistance to polymyxin (Petrou et al., 2016).

Among the 30 *P. mirabilis* strains, 15 contained the Inc plasmid characteristic gene, *repC* of IncQ1α. Analysis of Inc plasmid characteristic genes in 126 public databases of *P. mirabilis* revealed the presence of *repC* genes. A combined analysis of all *repC* genes from these 15 strains and public data showed high similarity, with an overall mean genetic distance of 0.006 ± 0.001 across the 15 sequences in this study and the 24 publicly available ones.

Analysis of whole genome data from 2 strains and 126 public datasets revealed that both strains isolated in this experiment contain structural genes of type-F plasmids, such as *TraV*, *TraU*, *TraN*, *TraL*, *TraK*, *TraI*, *TraH*, *TraG*, *TraF*, *TraE*/*GumN*, and *TraA*. Among all 128 datasets, the proportion of structural genes in type-F plasmids is relatively high, with the majority of structural genes ranging over 40%. The other Inc structural genes that mediated the conjugation transfer, such as *MobC*, *trbB*, *trbD*, and so on, mainly account for less than 5%. Analysis of type-F plasmid structural genes in 128 strains showed that all contained these genes, but their compositions varied significantly. The 128 strains exhibited 48 patterns, with no correlation found.

An equilibrium in the transmission process may favor the persistence of *P. mirabilis*. The *repC* gene was primarily associated with IncQ1α plasmids, along with structural genes of other F-type plasmids. Evidence includes the pattern indicating no regularity. Genes such as *TraV*, *TraU*, *TraN*, *TraL*, *TraK*, *TraI*, *TraH*, *TraG*, *TraF*, *TraE*/*GumN*, and *TraA* have higher expression levels. The *repC* expresses only one type. The lack of regularity suggested the multi-source origins, while the high expression levels of F-type plasmid structural genes indicated a significant prevalence. The *repC* gene has a relatively close genetic distance. More extensive epidemiological studies to understand its severity were needed.

## 5 Conclusion

The *P. mirabilis* isolates derived from pigs in Kunming were predominantly positive for the *repC* gene associated with the IncQ1α plasmid and also carried structural genes of the F-type plasmid. This trend was similarly observed in the 126 publicly available genome datasets used for comparison. *P. mirabilis* may maintain this plasmid composition to achieve a balance in its propagation and survival.

## Data Availability

The datasets presented in this study can be found in online repositories. The names of the repository/repositories and accession number(s) can be found in the article/Supplementary material.

## Footnotes

^1^ https://nanoporetech.com/

^2^ https://rast.nmpdr.org/rast.cgi

^3^ https://pubmlst.org/

^4^ https://card.mcmaster.ca/

^5^ https://blast.ncbi.nlm.nih.gov

## Appendix

**Table A1:** Antimicrobial category.
