# Supplementary material for: Prevalence and characterization of IncQ1α-mediated multi-drug resistance in Proteus mirabilis Isolated from pigs in Kunming, Yunnan, China
Source: Front Microbiol. 2025 Jan 9;15:1483633. doi: 10.3389/fmicb.2024.1483633 (PMC11754265; doi:10.3389/fmicb.2024.1483633)
Supplement: Supplementary file 2 [file Data_Sheet_1.DOCX]

Phylogenetic relationships of the main resistance genes (*vanG*, *rsmA*, *PBP3*, *KpnH*, *KpnF*, *gyrB*, *EF-TU*, *CRP*, *catA4*, *ArnT*, and *adeF*), including those from 2 strains in this experiment and 16 representative various region strains from public data.

Among these 18 *P*. *mirabilis* strains, the evolutionary trees were constructed by utilizing genes including (*vanG*, *rsmA*, *PBP3*, *KpnH*, *KpnF*, *gyrB*, *EF-TU*, *CRP*, *catA4*, *ArnT*, and *adeF*). However, no distinct correlation were discovered.

Dendrogram of *vanG* among 18 strain *P*. *mirabilis.*

Dendrogram of *rsmA* among 18 strain *P*. *mirabilis.*

Dendrogram of *PBP3* among 18 strain *P*. *mirabilis.*

Dendrogram of *KpnH* among 18 strain *P*. *mirabilis.*

Dendrogram of *KpnF* among 18 strain *P*. *mirabilis.*

Dendrogram of *gyrB* among 18 strain *P*. *mirabilis.*

Dendrogram of *EF-TU* among 18 strain *P*. *mirabilis.*

Dendrogram of *CRP* among 18 strain *P*. *mirabilis.*

Dendrogram of *catA4* among 18 strain *P*. *mirabilis.*

Dendrogram of *ArnT* among 18 strain *P*. *mirabilis.*

Dendrogram of *adeF* among 18 strain *P*. *mirabilis.*
